# VE-cadherin RGD motifs promote metastasis and constitute a potential therapeutic target in melanoma and breast cancers

**DOI:** 10.18632/oncotarget.13832

**Published:** 2016-12-09

**Authors:** Rubén A. Bartolomé, Sofía Torres, Soledad Isern de Val, Beatriz Escudero-Paniagua, Eva Calviño, Joaquín Teixidó, J. Ignacio Casal

**Affiliations:** ^1^ Department of Cellular and Molecular Medicine, Centro de Investigaciones Biológicas (CIB-CSIC), Ramiro de Maeztu, Madrid, Spain

**Keywords:** VE-cadherin, RGD motif, metastasis, melanoma, breast cancer

## Abstract

We have investigated the role of vascular-endothelial (VE)-cadherin in melanoma and breast cancer metastasis. We found that VE-cadherin is expressed in highly aggressive melanoma and breast cancer cell lines. Remarkably, inactivation of VE-cadherin triggered a significant loss of malignant traits (proliferation, adhesion, invasion and transendothelial migration) in melanoma and breast cancer cells. These effects, except transendothelial migration, were induced by the VE-cadherin RGD motifs. Co-immunoprecipitation experiments demonstrated an interaction between VE-cadherin and α2β1 integrin, with the RGD motifs found to directly affect β1 integrin activation. VE-cadherin-mediated integrin signaling occurred through specific activation of SRC, ERK and JNK, including AKT in melanoma. Knocking down VE-cadherin suppressed lung colonization capacity of melanoma or breast cancer cells inoculated in mice, while pre-incubation with VE-cadherin RGD peptides promoted lung metastasis for both cancer types. Finally, an *in silico* study revealed the association of high VE-cadherin expression with poor survival in a subset of melanoma patients and breast cancer patients showing low CD34 expression. These findings support a general role for VE-cadherin and other RGD cadherins as critical regulators of lung and liver metastasis in multiple solid tumours. These results pave the way for cadherin-specific RGD targeted therapies to control disseminated metastasis in multiple cancers.

## INTRODUCTION

Metastasis, the final step of malignant transformation, is a complex process with numerous distinct and sequential steps. Organ-specific metastasis depends on different molecules, including growth factors, receptors, proteases, chemokines and cellular adhesion molecules, such as cadherins and integrins [1, 2]. Recently, we described the presence of tripeptide RGD motifs in 7D cadherins, cadherin-17 (CDH17; also LI-cadherin) and cadherin-16, and type II cadherins, such as vascular endothelial (VE)-cadherin (CD144), KSP-cadherin (CDH6) and cadherin 20 (CDH20) [3]. CDH17 activates the α2β1 integrin pathway through specific RGD motifs to promote cell adhesion, proliferation and liver colonization in colorectal cancer metastasis [3, 4]. A direct interaction between CDH17 and α2β1 integrin was demonstrated using various methods, such as affinity chromatography, soluble binding of cadherin ectodomains and cell adhesion to RGD-containing cadherin domains [3].

VE-cadherin is a type II cadherin that contains RGD motifs within different domains (e.g. 1, 2 or 3) in many mammals and birds, but not in rodents or dogs (Supplementary Data Figure 1). In primates, the VE-cadherin sequence displays two RGD motifs in cadherin domains 2 and 3 (Supplementary Data Figure 1). VE-cadherin is mainly expressed in endothelial cells, where it plays a central role in vascular integrity and permeability and promotes homotypic cell-to-cell adhesion [5]. Intracellular association to β- and γ-catenin (plakoglobin) promotes binding to the actin cytoskeleton [6]. Mice deficient in VE-cadherin, or expressing truncated VE-cadherin, die in mid-gestation from severe vascular defects, involving endothelial apoptosis and disrupted survival signalling pathways [7]. VE-cadherin has been used as a target to control tumour angiogenesis *in vivo* [8]. It is also expressed in Ewing sarcoma [9], highly aggressive cutaneous melanomas [10] and it is involved in vasculogenic mimicry (the ability to form novel blood vessel-like structures) in uveal melanomas [11]. VE-cadherin is also expressed in a subset of acute lymphoblastic leukemia cells, where contributes to cell survival [12], and in a subset of cancer stem cells CD133^+^ in osteosarcoma, ovarian cancer and glioblastoma, contributing to vasculogenic mimicry by VEGF-independent tumour cell differentiation [13].

VE-cadherin enhances the capacity of mouse mammary tumour cells to proliferate and adhere to endothelial cells [14, 15]. Its effects on cell proliferation have been commonly attributed to β-catenin release from the p120-catenin complex and induction of transcription of specific genes [16]. In addition, α2β1 integrin-mediated phosphorylation of VE-cadherin leads to the disorganization of the endothelial adherens junctions and facilitates transendothelial migration of breast cancer cells. This effect was mediated by the binding of α2β1 integrin to an unknown counterligand on endothelial cells [17]. Still, many aspects regarding signalling effects of VE-cadherin on the epithelial cancer cells and their effect on invasion and proliferation were still obscure.

Here, we investigated the role of VE-cadherin in metastasis progression of melanoma and breast cancers. We have identified that VE-cadherin activates α2β1 integrin through its RGD motifs, thereby using the integrin signalling pathway to promote adhesion, invasion and proliferation. We provide evidence that the VE-cadherin RGD motifs promote *in vivo* lung metastasis in melanoma and breast cancers. Moreover, a high expression of VE-cadherin in melanoma and breast cancer patients is associated to poor prognosis.

## RESULTS

### VE-cadherin expression is associated to metastatic melanoma and breast cancer cell lines

**Figure 1:**
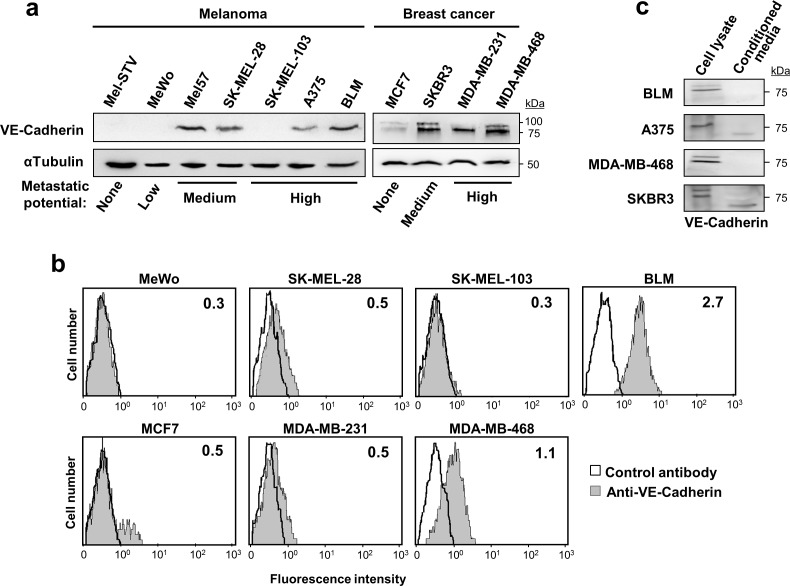
VE-cadherin is expressed in melanoma and breast cancer cell lines **a.** Protein lysates of the indicated melanoma and breast cancer cell lines were resolved by SDS-PAGE and subjected to western blot using anti-VE-cadherin. Anti-α-tubulin was used to assess total protein content. **b.** Flow cytometry analysis showing the surface expression of VE-cadherin on the indicated cancer cells. Inside each panel, mean fluorescence intensity is indicated. **c.** VE-cadherin expression was assessed by western blot in cell lysates and 48-h conditioned media of the indicated melanoma and breast cancer cell lines.

We analysed VE-cadherin expression and its role in metastasis in a panel of 8 melanoma and 4 breast cancer cell lines (Figure 1a). Compared to non-invasive MCF7, VE-cadherin overexpression was observed in metastatic breast cancer cells (SKBR3, MDA-MB-231 and MDA-MB-468) and metastatic melanoma cell lines (BLM, A375, SK-MEL-28 and Mel57) except SK-MEL-103. VE-cadherin was absent in Mel-STV melanocytes and in the poorly metastatic MeWo cell line. Interestingly, breast cancer cells exhibited two forms of VE-cadherin while melanoma cells showed only one, probably due to cell-specific differences in glycosylation. Flow cytometry analysis confirmed a correlation between cell surface expression of VE-cadherin and total expression observed by western blot (Figure 1b). Secretion of the VE-cadherin ectodomain was detected in A375 melanoma cancer cells and SKBR3 breast cancer cells (Figure 1c). In any case, 75% of the cadherin was found in the cell lysate, indicating that VE-cadherin signalling would be mainly from the cell surface. For the remaining studies, two representative metastatic cell lines for each cancer type —BLM and A375 for melanoma and MDA-MB-468 and SKBR3 for breast cancer—were used.

### RGD peptides in VE-cadherin increase adhesion, proliferation and invasion in melanoma and breast cancer cells

**Figure 2:**
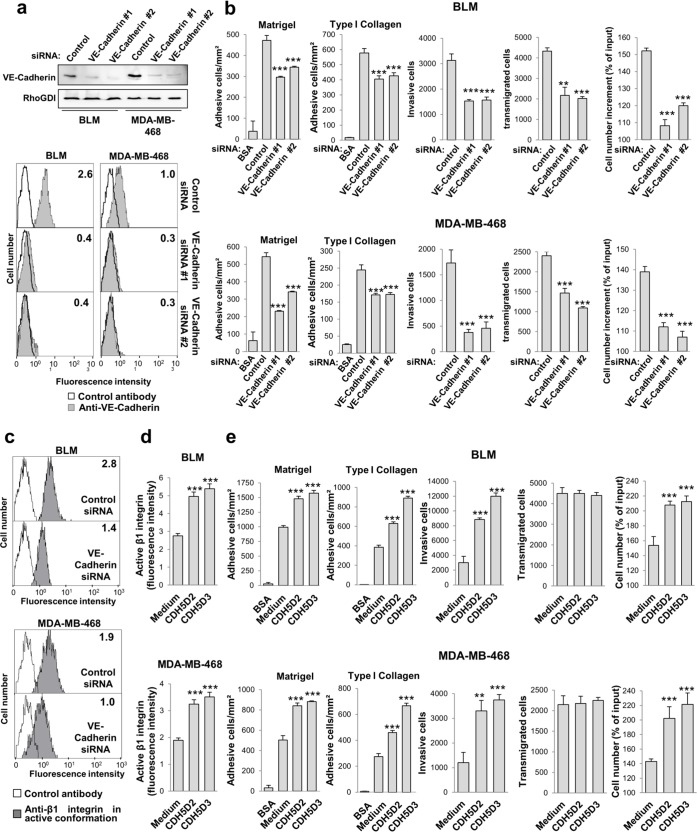
VE-cadherin RGD motifs promote cell adhesion, invasion and proliferation **a.** BLM and MDA-MB-468 cells were transfected with one of two distinct siRNAs against VE-cadherin or with a control siRNA. VE-cadherin expression was assessed by western blot and flow cytometry. **b.** Transfectants were subjected to adhesion onto Matrigel or type I collagen, invasion through Matrigel, endothelial transmigration and MTT assays to determinate cell proliferation. Cell adhesion, invasion, transmigration or proliferation was significantly inhibited by VE-cadherin knock-down (** *P* < 0.01, *** *P* < 0.001). **c.** Transfectants were analysed by flow cytometry to determine the expression of high-affinity β1 integrin conformation. **d.** BLM and MDA-MB-468 cells were exposed to 9-mer peptides containing the RGD motifs and flanking sequences from VE-cadherin domain 2 or domain 3 (CDH5D2 or CDH5D3, respectively) and subjected to flow cytometry assays to assess the amount of β1 integrin in high affinity conformation. β1 integrin was significantly activated by exposition to the peptides (*** *P* < 0.001). **e.** Same as **b.** but in the presence of the CDH5D2 or CDH5D3 peptide. Cell adhesion, invasion or proliferation was significantly enhanced by peptide addition (** *P* < 0.01, *** *P* < 0.001).

Next, we investigated whether VE-cadherin silencing affects the pro-metastatic properties of melanoma and breast cancer cells. Cancer cells transfected with either one of two distinct VE-cadherin siRNAs exhibited a clear reduction of VE-cadherin expression by western blot and flow cytometry (Figure 2a and Supplementary Data Figure 2a). VE-cadherin silencing led to a significant decline in the cellular responses of proliferation, invasion, transendothelial migration and adhesion (Figure 2b and Supplementary Data Figure 2b). Flow cytometry analysis using a monoclonal antibody specific for the high-affinity conformation of β1 integrin demonstrated that VE-cadherin silencing in the four cell lines significantly reduced the high affinity conformation (Figure 2c and Supplementary Data Figure 2c), but had no effect on the total level of β1 integrin expression (Supplementary Data Figure 3), indicating that VE-cadherin regulates the affinity but not the expression of β1 integrin. We thus investigated whether these effects are mediated through the VE-cadherin RGD motifs.

Addition of VE-cadherin RGD peptides significantly increased the activation of β1 integrin (Figure 2d), as well as the adhesion, invasion and proliferation responses in melanoma and breast cancer cells (Figure 2e). With respect to the adhesion response, VE-cadherin increased cell binding to collagen type I and Matrigel (Figure 2e). In contrast, the RGD peptides did not affect the transendothelial migration capacity of tumour cells across an HUVEC endothelial cell monolayer, perhaps because the abundant expression of VE-cadherin in HUVEC tampers the peptide effects, or perhaps because the RGD motif does not participate in homotypic cell-to-cell interactions [3]. Collectively, these data indicate that the VE-cadherin RGD motifs play a major role in cell adhesion, invasion and proliferation in melanoma and breast cancer cells, which correlate directly with β1 integrin activation. The effect of VE-cadherin on transendothelial migration is likely mediated by homotypic interactions.

### VE-cadherin protein complexes are linked to integrin signalling and cell adhesion

**Figure 3:**
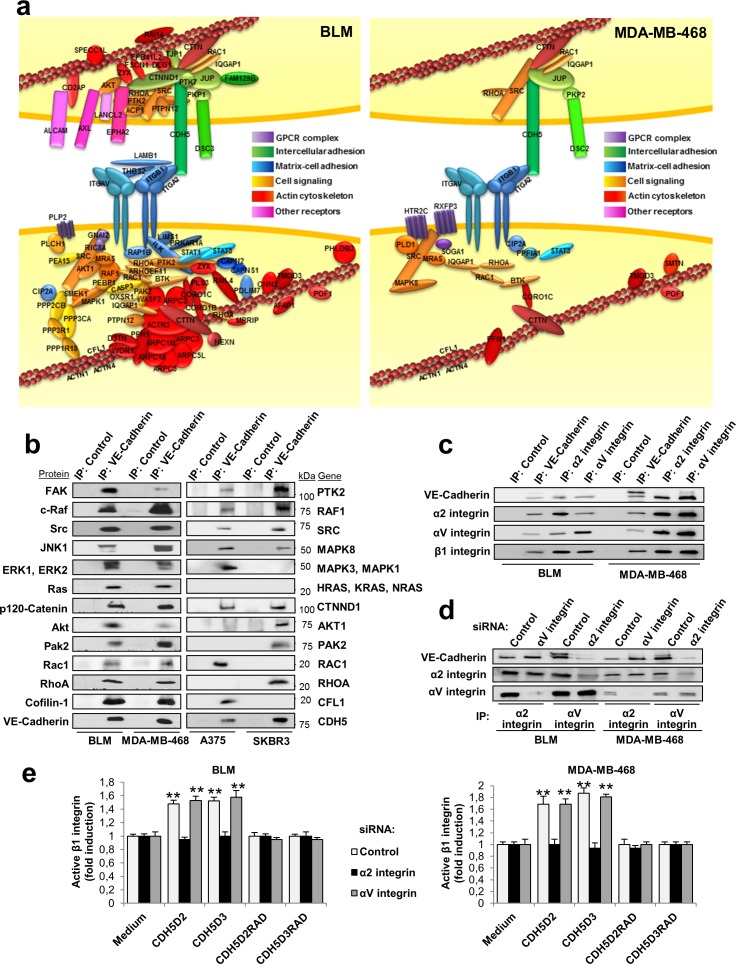
Protein networks identified by proteomic analyses after VE-cadherin immunoprecipitation **a.** Cell lysates from BLM and MDA-MB-468 cells were immunoprecipitated using anti-VE-cadherin or control antibodies. Immunoprecipitates were resolved by SDS-PAGE, divided in three fractions and digested with trypsin. The resulting peptides were analysed by nanoLC-MS/MS. The diagram displays the proteins that were determined to specifically bind to VE-cadherin by bioinformatic analyses and data mining. **b.** Cell lysates from the indicated cells lines were immunoprecipitated as before, resolved by SDS-PAGE and analyzed by western blot using the indicated antibodies. **c.** Analysis by western blot of cell lysates from BLM and MDA-MB-468 immunoprecipitated using anti-VE-cadherin, anti-α2 integrin, anti-αv integrin or control antibodies. **d.** BLM or MDA-MB-468 cells were transfected with siRNAs against α2 integrin, αv integrin or control siRNAs. Cell lysates from the transfectants were subjected to immunoprecipitation using the indicated antibodies and analyzed by western blot. **e.** The same siRNA transfectants were exposed to the indicated peptides. β1 integrin was significant activated by exposition to the peptides (** *P* < 0.01).

In contrast to CDH17, VE-cadherin contains a large cytoplasmic domain engaged in signal transmission. To characterize the protein-interaction network of VE-cadherin, we used immunoprecipitation and mass spectrometry. After bioinformatic analysis, we identified 79 proteins that co-immunoprecipitated specifically with VE-cadherin in BLM melanoma cells, and 24 proteins in MDA-MB-468 breast cancer cells (Supplementary Data Figure 4a and Supplementary Table 2). The VE-cadherin interaction network in both cell types contained α2β1 integrin, αV integrin and other proteins mainly involved in cell-matrix adhesion, cell-cell adhesion, cell signalling and the actin cytoskeleton (Figure 3a and Supplementary Data Figure 4b). On the other hand, we confirmed the interaction of VE-cadherin with p120-catenin and plakoglobin through the cytoplasmic domain [16]. In BLM melanoma cells, additional interactions with ALCAM, associated with high invasiveness and aggressiveness in melanomas, were also observed [18]. Among other VE-cadherin-associated proteins involved in cell signalling, survival and proliferation, we identified FAK, SRC, Ras, AXL, EPHA2, AKT, STAT1, STAT3, JNK and ERK kinases (Figure 3a). Although the number of interacting proteins was larger in melanomas than in breast cancer cells, many identified proteins were similar and functionally equivalent in both cancer types. Therefore, VE-cadherin has homologous protein networks in melanoma and breast cancer. Interaction between VE-cadherin and integrin may occur in *trans* (Figure 3a) and/or *cis* (Supplementary Data Figure 4c), as experimental data are compatible with both options. VE-cadherin-associated proteins were confirmed by western blot in the four cell lines, with only a few exceptions (lack of Ras in A375 and SKBR3 cells) (Figure 3b).

The association of VE-cadherin with α2β1 integrin, and the presence of αV integrin in the complex, were confirmed by combined co-immunoprecipitation and silencing studies (Figure 3c, 3d). α2β1 integrin interacts with VE-cadherin, as α2 integrin silencing caused the loss of VE-cadherin in the immunoprecipitate (Figure 3d and Supplementary Data Figure 5). In contrast, αV silencing did not change the levels of VE-cadherin. Still, we cannot exclude that αV associates with β1, or other integrin β subunits, and clusters together with the α2β1 integrin. To investigate any potential effects of αVβ1 integrin, we carried out αV and α2 integrin silencing in combination with RGD peptide stimulation in BLM melanoma and MDA-MB-468 breast cancer cell lines (Figure 3e). Activation of β1 integrin by RGD peptides was blocked by α2 integrin silencing but not by αV silencing in both cell lines (Figure 3e). As expected, mutant RAD peptides failed to activate β1 integrin. In summary, the VE-cadherin interacting network consisted of α2β1 integrin and a large number of proteins from the integrin signalling pathway, actin cytoskeleton and cell adhesion, in addition to p120 catenin and plakoglobin.

### VE-cadherin signalling is involved in cell proliferation, invasion and endothelial transmigration in melanoma and breast cancer cells

**Figure 4:**
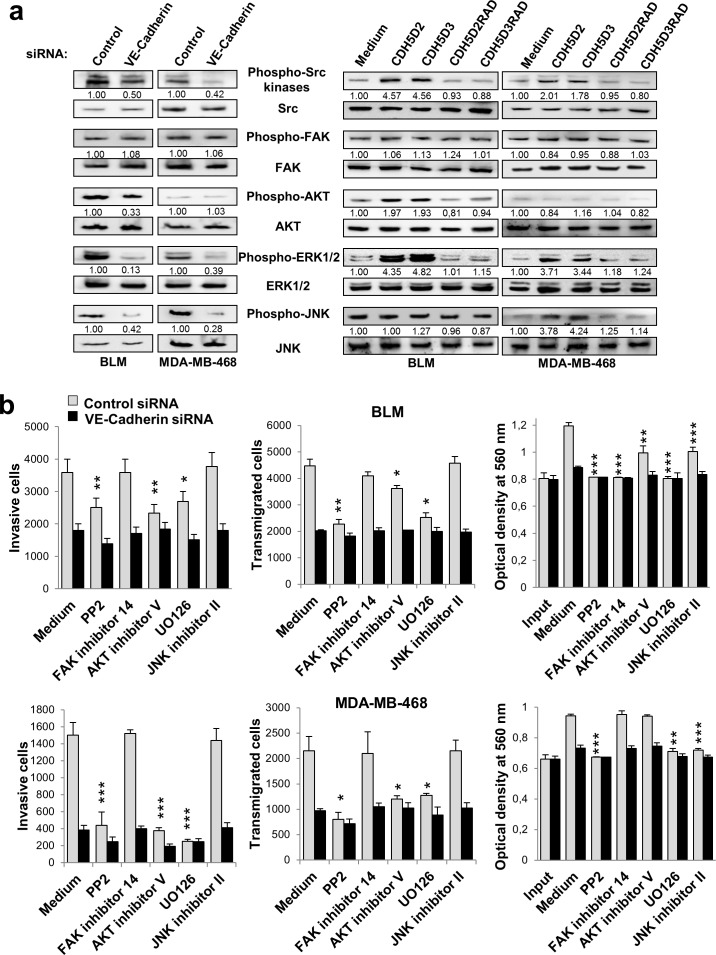
VE-cadherin regulates the activation of signalling proteins involved in cell invasion, transmigration and proliferation Cancer cells were transfected with **a.** VE-cadherin or control siRNAs or **b.** exposed to the indicated peptides. Cell lysates were analyzed by western blot using the indicated antibodies. Bands were quantified using MultiGauge software. **b.** siRNA transfectants were tested in cell invasion, endothelial transmigration and MTT assays in presence of the indicated chemical inhibitors.

We next investigated downstream mediators of VE-cadherin-α2β1 integrin signalling pathway in melanoma and breast cancer cells using specific inhibitors. Integrin signalling mediates different aspects of cancer progression, including invasion and proliferation [19]. After VE-cadherin silencing, phosphorylation of SRC, ERK and JNK was significantly decreased in both cell lines, and that of AKT in BLM melanoma cells (Figure 4a). Whereas enhanced integrin signalling was observed after incubation with the RGD peptides, no changes in phosphorylation were observed after using the respective RAD mutant sequences (Figure 4a). We then tested specific inhibitors for SRC, FAK, AKT, ERK/MEK and JNK to examine their effects on the metastatic properties, using either VE-cadherin-silenced or mock-silenced cells. None of these inhibitors affected β1 integrin activation or cell adhesion (Supplementary Data Figure 6). However, significant effects were observed for invasion, transendothelial migration and proliferation (Figure 4b). SRC (PP2), AKT and ERK/MEK (UO126) inhibitors provoked a clear decrease in the invasive and transendothelial migration capacity of scrambled melanoma and breast cancer transfectants, similar to those achieved by VE-cadherin-silenced counterparts. No effects were observed with the FAK or the JNK inhibitors in invasion or transendothelial migration. In contrast, AKT activation in breast cancer cells was important in both, invasion and transmigration, by mechanisms VE-cadherin-independent (Figure 4a). A decrease in proliferation was observed after using PP2, UO126 and JNK inhibitors in both cancer types. Proliferation of BLM melanoma cell was also sensitive to the FAK inhibitor in a mechanism VE-cadherin-independent (Figure 4a). Inhibitors did not affect significantly to VE-cadherin-silenced cell proliferation. Collectively, these results indicate that VE-cadherin promotes invasion and transendothelial migration by activation of the α2β1 integrin pathway through SRC and ERK. JNK activation was also implicated in cell proliferation.

### VE-cadherin promotes metastatic colonization in lung through its RGD motifs

**Figure 5:**
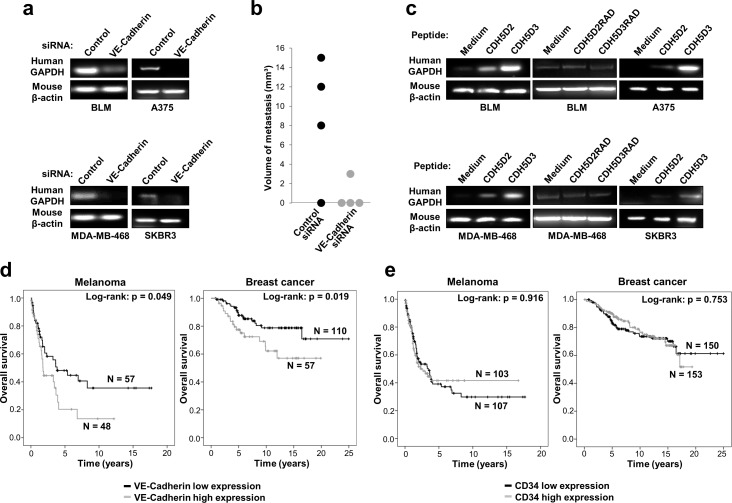
VE-cadherin expression is associated with lung metastasis in mouse models and poor survival in melanoma and breast cancer patients **a.** BLM, A375, MDA-MB-468 or SKBR3 cells transfected with VE-cadherin or control siRNAs were inoculated intravenously in nude mice. After 4 days, mice were euthanized and lung mRNAs were isolated and subjected to RT-PCR to amplify human GAPDH. As a control, mouse β-actin was also amplified. **b.** BLM cells transfected with the indicated siRNAs were inoculated intravenously in nude mice (*n* = 4). After 35 days, mice were euthanized and macroscopic lung metastases were measured. **c.** Cancer cells were inoculated in nude mice together with a peptide containing the RGD motifs (CDH5D2 or CDH5D3) or an irrelevant RAD sequence (CDH5D2RAD or CDH5D3RAD), as indicated. After 4 days, lung mRNAs were isolated and analysed by RT-PCR as before. **d.** Significant association of VE-cadherin expression with shorter survival of melanoma and breast cancer patients was found using the log-rank test. **e.** CD34 expression is not associated with poor survival in melanoma and breast cancer patients as found using the log-rank test.

We next investigated the role of VE-cadherin and its RGD motifs in melanoma and breast cancer metastasis *in vivo*. We hypothesized that pre-incubation of the cells with the peptides should activate α2β1 integrin and, consequently, the pro-metastatic activity of the cells. For this study, we intravenously inoculated VE-cadherin knockdown or wild-type control cells in mice and investigated whether lung metastasis was induced by testing for the presence of human *GAPDH* in lung tissue. Mice were tested at 96 h post-inoculation to avoid non-specific cell binding to the lung. Lungs obtained from mice inoculated with VE-cadherin-silenced cells showed a much lower amount of the human housekeeping *GAPDH* gene than those inoculated with control cells (Figure 5a). In addition, lung metastasis after 5 weeks was significantly decreased in mice inoculated with VE-cadherin silenced BLM melanoma cells, compared to mice inoculated with control siRNA transfectants (Figure 5b). To confirm the pro-metastatic role of the VE-cadherin RGD motifs, we pre-incubated the cells with 9-mer peptides containing the RGD motifs in domains 2 and 3 prior to mouse inoculation. After 96 h, we noticed a significant increase in the lung colonization of melanoma and breast cancer cells, especially after using domain 3 RGD (Figure 5c). This increase in metastatic colonization was lost when the RGD peptides were replaced by the inactive RAD mutant peptides (Figure 5c). Altogether, these experiments revealed an important role of VE-cadherin with involvement of the RGD motifs in the lung metastasis of melanoma and breast cancer cells.

### Impact of VE-cadherin expression in patient survival

Little is known about the impact of VE-cadherin expression on the clinical outcome of melanoma and breast cancer patients. To explore the prognostic and clinical value of VE-cadherin in patients, we performed *in silico* analysis of overall survival using GEO datasets. As VE-cadherin is also expressed by endothelial cells, which are usually found in the tumours, we tested only samples with low expression of CD34, a marker of endothelial cells, to discard tumours with an excessive angiogenesis that might lead to confusing results. In melanoma and breast cancer, CD34 *low* patients with high expression of VE-cadherin exhibited a significant reduction in their survival time (log-rank *P* = 0.049, log-rank *P* = 0.019, respectively) (Figure 5d). Thus, high VE-cadherin expression was associated in a statistically significant manner with poorer overall survival for melanoma and breast cancer patients. In contrast, we did not find association between CD34 expression and survival (Figure 5e). These results support that the correlation between VE-cadherin expression and overall survival in melanoma and breast cancer patients is not due to high vascularization of the tumours.

## DISCUSSION

**Figure 6:**
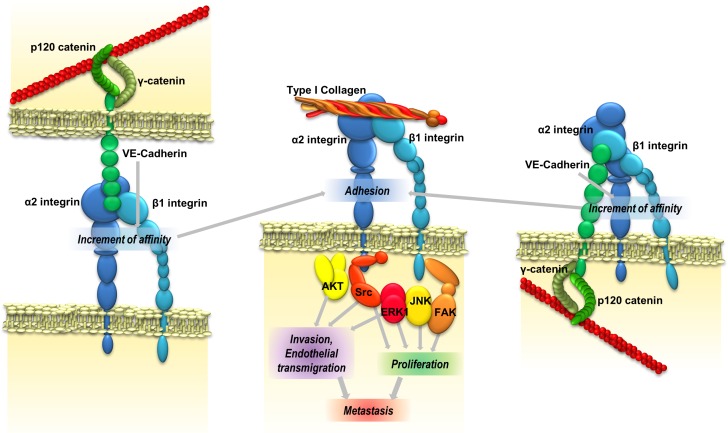
VE-cadherin promotes metastatic colonization VE-cadherin RGD motifs might interact with α2β1 integrin either inter- or intracellularly, thereby increasing its affinity for ligands in the extracellular matrix and leading to an increase of cell adhesion and signalling activation proteins. Integrin activation increases the ability of cancer cells to invade and proliferate, and consequently their metastatic capacity.

Here, we have demonstrated that VE-cadherin plays a major role in the induction of pro-metastatic properties—adhesion, invasion and proliferation—in different melanoma and breast cancer cell lines. We provide different pieces of evidence that the functional effects were mediated by binding of the VE-cadherin RGD motifs to α2β1 integrin. A model of the signalling pathway is represented in Figure 6. These results confirm the relevance of RGD cadherins in the development of cancer metastasis in multiple tumours.

However, beyond cell specificity, there are important differences between RGD cadherins from a mechanistic point of view. For instance, VE-cadherin has two RGD motifs, which are located in domains 2 and 3 and are far from the cell surface, while CDH17 has one RGD, which is located in domain 7 and is quite close to the cell membrane. Moreover, while VE-cadherin contains a functional cytoplasmic domain [16], CDH17 contains a very small cytoplasmic domain not adequate for intracellular signalling. CDH17-mediated integrin activation was observed to mainly affect adhesion and proliferation in colorectal cancer [4], while VE-cadherin RGD motifs also affected invasion in melanoma and breast cancer cells. Whereas metastatic colorectal cancer cells lose their capacity to bind collagen type I and preferably bind collagen IV after CDH17 activation of α2β1 integrin [3], melanoma and breast cancer cells retained their binding capacity to collagen I. These differences in collagen-binding capacities between both cadherins are probably due to the fact that liver parenchyma is mainly composed of collagen type IV, while lung parenchyma mostly contains type I and III collagen [20]. This is, they are related to the organ-specificity in the final metastatic colonization. *In vivo* experiments showed that VE-cadherin silencing decreases lung colonization, while the addition of VE-cadherin RGD peptides potentiate the metastatic colonization of the lung.

It is interesting that many animal models used in cancer research, such as rodents or dogs, lack RGD motifs in their VE-cadherin sequence (Supplementary Data Figure 1). In contrast, the VE-cadherin sequence in most primates contains two RGD domains, implicating a relatively late trait acquisition that might provide some evolutionary advantage. Metastatic cells might take advantage of this evolutionary trait to improve their proliferation and invasion capacity. The lack of RGD motifs in rodent cadherins highlights that caution should be used with direct extrapolations of mouse cancer metastasis models to humans, but it also implies the existence of additional ways for metastatic colonization.

Our experiments indicate that VE-cadherin signalling does not involve αVβ1 integrin, which has been considered to be an RGD receptor [21], nor αVβ3, which was not detected. In any case, the presence of αV in the complex deserves further investigation. In addition, we observed an interaction of VE-cadherin with p120-catenin and plakoglobin, but not with β-catenin. Therefore, VE-cadherin in cancer cells keeps its cytoskeleton binding capacity (actin-binding) but does not seem to require the interaction with β-catenin for SRC or FAK activation, as it does in endothelial cells [16]. In cancer cells, SRC, ERK and JNK activation would mediate the VE-cadherin/integrin signalling. VE-cadherin effects on proliferation of metastatic mammary cancer cells, which were previously attributed to the TGFβ pathway [14], could also be explained with the outside-in α2β1 integrin activation and the subsequent activation of the SRC, ERK and JNK pathways. Still, we cannot rule out that VE-cadherin might promote TGFβ activation through other indirect interactions. A correlation between higher expression of α2β1 integrin and higher invasiveness was previously observed in breast cancer cells [17]. RGD binding might additionally promote tyrosine phosphorylation of VE-cadherin in endothelial cells, which is mediated by α2β1 integrin in invasive breast cancer cells [17]. In addition, tyrosine phosphorylation of VE-cadherin initiates the β-catenin dissociation from the p120-catenin complex to disrupt the endothelial barrier [22]. Since VE-cadherin is phosphorylated in cancer cells, it might explain why β-catenin was absent from our interaction data.

VE-cadherin is expressed also in endothelial cells and the *in silico* meta-analysis was carried out using RNA expression data from complete tumour tissues, which contain not only epithelial cells but stromal and endothelial cells. Therefore, more VE-cadherin could correlate with more angiogenesis, which in turn could finally affect the patient's survival. To clarify this point and to avoid a misinterpretation, we classified the tumours according to the expression of the endothelial marker CD34 in order to discard those tumours with high angiogenesis and vessels content from the analysis. While no association of CD34 expression with survival was observed, those melanoma and breast cancer patients who expressed high levels of VE-cadherin and low levels of CD34 showed a clear association with worst prognosis.

In summary, these studies confirm the key role of VE-cadherin and their RGD motifs in facilitating integrin activation and metastatic colonization in multiple cancer types. Interestingly, RGD motif was not required for homotypic interaction or transendothelial migration. The dissociation of the homotypic cell-cell interaction from the RGD-integrin signalling might avoid interfering with vascular stability. The enhancement of melanoma and breast cancer lung metastasis by the VE-cadherin RGD motif highlights the value of cadherin-specific RGD targeting as a novel therapeutic strategy for multiple types of cancer and metastasis. This strategy might be especially relevant for aggressive melanomas (including uveal melanoma) and breast cancer subtypes with poor prognoses, which are particularly resistant to current therapies.

## MATERIALS AND METHODS

### Cell lines, culture and reagents

Cells were cultured in Dulbecco's modified Eagle medium (DMEM) (Invitrogen) or RPMI (MeWo cells) containing 10% foetal calf serum (Invitrogen) and antibiotics at 37°C in a 5% CO_2_-humidified atmosphere. Mel57 and SK-MEL-103 were gifts from Dr M. Soengas, MeWo and SK-MEL-28, from Dr P. Real (CNIO, Madrid, Spain), BLM cells, from Dr G. van Muijen (University Hospital, Nijmegen, The Netherlands), Mel-STV, from Dr. R. Weinberg (MIT, Cambridge, MA, USA) and MDA-MB-468 and SKBR3, from Drs A Aranda and A Villalón, respectively (IIBB, Madrid, Spain). Cell lines A375, MDA-MB-231, MCF7 and RKO cells were purchased from the ATCC and passaged fewer than 6 months after purchase for all the experiments.

The following siRNAs were used for expression silencing: VE-cadherin, SASI-Hs01_00100751 or EHU131141 (Sigma) (assays were carried out with each one of the two siRNAs, with consistently equivalent results), integrin αV, SASI_Hs01_00220507 (Sigma), and integrin α2, SASI_Hs01_00123982 (Sigma). These siRNAs were transfected in tumour cells using JetPrime reagent (PolyPlus). In each transfection, an aliquot of cells was lysed and analyzed by western blot to assess VE-cadherin expression silencing. Antibodies used are listed in the Supplementary Data Table S2. Nine-amino-acid peptides containing the RGD motifs from VE-cadherin were chemically synthesized in a Focus XC peptide synthesizer (AAPPTec, KY, USA). The peptides, named according to the cadherin domain where the RGD motif is found, were CDH5D2 (QGLRGDSGT, residues 233-241) and CDH5D3 (SILRGDYQD, residues 296-304). As a negative control, peptides with the RGD motif mutated into an irrelevant RAD sequence—CDH5D2RAD or CDH5D3RAD—were used. The following inhibitors were used: UO126 and AKT inhibitor V (Calbiochem), PP2 (Sigma-Aldrich), JNK inhibitor II (Merck Chemicals) and the FAK inhibitor 14 (Santa Cruz Biotechnology).

### Flow cytometry

Cells were detached in PBS with 2 mM EDTA, washed and resuspended in PBS with human gamma globulin (10 μg ml^−1^) in presence of primary antibodies (10 μg ml^−1^) and incubated for 40 min. After washing, cells were incubated in the same medium with Alexa 468-labelled anti-mouse IgG antibodies (DAKO A/S) for 30 min. Data were analysed in a Coulter Epics XL cytofluorometer.

### Cell adhesion and proliferation

Cell adhesion and proliferation assays were carried out as previously described [3].

### Cell invasion assays

For invasion, 3 × 10^4^ cells were resuspended in serum-free DMEM and loaded onto 8-μm pore-size filters coated with 35 μL of a 1:3 dilution of Matrigel on Transwells (Costar). The lower compartments of the invasion chambers were filled with DMEM with 5% serum, while inhibitors were added to the upper compartments. After 40 h of incubation at 37°C, non-invading cells were removed from the upper surface of the filter, and invasive cells were fixed with 4% paraformaldehyde (Sigma-Aldrich), stained with crystal violet and counted under a microscope.

### Transendothelial migration assays

HUVEC cells (5 × 10^4^) were plated on 8-μm pore-size filter coated with fibronectin (20 μg ml^−1^; Invitrogen), and confluent monolayers were incubated 16 h before the assay with TNF-β (15 ng ml^−1^, R&D Systems). Tumour cells (3 × 10^4^) were resuspended in serum-free DMEM, loaded onto the filters and incubated 24 h. After non-migrating cells and endothelial cells had been removed, migrating cells were fixed, stained and counted as for invasion assays.

### Western blot and immunoprecipitation

Cells were detached, washed and lysed with proteases and phosphatase inhibitors in lysis buffer (1% Igepal, 100 mM NaCl, 2 mM MgCl_2_, 10% glycerol, 50 mM Tris-HCl). Conditioned media were concentrated 10 times using Vivaspin 15R (Sartorius). Protein extracts were separated in SDS-PAGE and transferred to nitrocellulose membranes, which were incubated with primary antibodies followed by incubation with an HRP-coupled secondary antibody (Thermo Scientific) and with SuperSignal West Pico Chemiluminescent Substrate (Thermo Scientific). Densitometry analysis was performed with Quantity One software (Bio-Rad). For immunoprecipitation, cells were lysed as before, and 500 μg of lysate were incubated with 100 μL of protein G sepharose to avoid unspecific protein binding, followed by incubation with the indicated antibodies and protein G sepharose beads (Sigma-Aldrich). After washing, samples were boiled and loaded on SDS-PAGE gels for western blot analysis and mass spectrometry.

### Mass spectrometry of immunoprecipitated proteins

For proteomic analysis, 10 mg of cell lysates were immunoprecipitated and the proteins were separated by SDS-PAGE, which were then divided in three slices for in-gel digestion with trypsin. Proteins were identified by mass spectrometry as previously described [4]. Identified peptides were validated using Percolator algorithm with a q-value threshold of 0.01. Identified proteins were filtered by using the CRAPome tool to discard contaminant proteins [23].

### *In vivo* animal experiments

For lung metastasis, 1 × 10^6^ BLM, A375, SKBR3 or MDA-MD-468 cells in 0.1 ml PBS were injected in the tail vein of Swiss nude mice (Charles River). Short term colonization assays were performed as described previously [24]. In brief, mice were euthanized 4 days after inoculation, and RNA was isolated from lungs using TRIzol Reagent (Invitrogen) and retrotranscribed. The cDNA was subjected to 30 cycles of PCR with TaqDNA polymerase (Invitrogen) to amplify human GAPDH, a housekeeping gene. For siRNA-transfected cancer cells, 1 μL of the previous reaction was subjected to additional 25-cycle amplification. As a loading control, a 30-cycle amplification of murine β-actin was performed. For long term assays, mice were euthanized 5 weeks after inoculation, and macroscopic lung metastases were counted and measured.

### *In silico* prognostic studies

Independent external cohorts of patients were used for the prognostic study. The GSE65904 database, which contains a cohort of 214 frozen melanoma samples and the GSE7390 and GSE45255 databases with 198 and 139 breast cancer samples, respectively, were used. Data were normalized using cubic spline method (melanoma) or MAS5 bioconductor (breast cancer), downloaded, and z-score ratios were obtained by subtracting the average z-score for the cohort from each individual z-score and dividing the result by the standard deviation of the z-scores for the cohort, in order to remove technical variations between datasets. CD34, an endothelial marker, and VE-cadherin were independently assessed for each database. Negative and positive z-score were considered as low and high expression, respectively. Only samples with low CD34 expression were analysed for VE-cadherin expression to avoid those samples with more endothelial cells/angiogenesis. The prognostic value of VE-cadherin was assessed using Kaplan-Meier survival curves, which were plotted using IBM SPSS Statistics 2.0. Log-rank test P value was determined for differences in overall survival.

### Statistical analyses

Data were analysed by Student's t test (2 conditions) or by one-way ANOVA followed by Tukey-Kramer multiple comparison test (more than 2 conditions). Survival curves were plotted with Kaplan-Meier technique and compared with the log-rank test. The minimum acceptable level of significance was P < 0.05.

## SUPPLEMENTARY MATERIAL







## Abbreviations

CDH17: Cadherin 17
DMEM: Dulbecco's modified Eagle medium
EMT: Epithelial-to-mesenchymal transition
EDTA: Ethylenediaminetetraacetic acid
GAPDH: Glyceraldehyde 3-phosphate dehydrogenase
HRP: Horseradish peroxidase
HUVEC: Human umbilical vein endothelial cells
PBS: Phosphate-buffered saline
PCR: Polymerase chain reaction; medium
RPMI: Roswell Park Memorial Institute
scFv: Single-chain variable fragment
SDS-PAGE: sodium dodecyl sulphate polyacrylamide gel electrophoresis
siRNA: small interfering RNA
TGF-β: Transforming growth factor-β
TNF-β: Tumour necrosis factor-β
VE: vascular endothelial
