# Supplementary material for: VE-cadherin RGD motifs promote metastasis and constitute a potential therapeutic target in melanoma and breast cancers
Source: Oncotarget. 2016 Dec 9;8(1):215–27. doi: 10.18632/oncotarget.13832 (PMC5352113; doi:10.18632/oncotarget.13832)
Supplement: Supplementary file 1 [file oncotarget-08-215-s001.pdf]

VE-cadherin RGD motifs promote metastasis and constitute a potential therapeutic target in melanoma and breast cancers

Supplementary Material

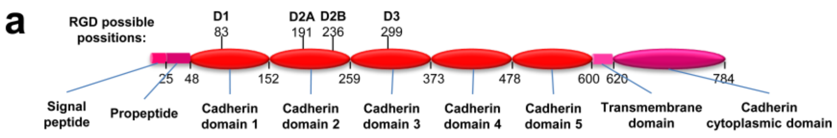

b

Class	Order	Species	Common name	RGD position
Mammalia	Didelphimorpha	*Monodelphis domestica*	Gray short-tailed opossum	-
	Dayuromorpha	*Sarcophilus harrisii*	Tasmanian devil	-
	Afrosoricida	*Chrysochloris asiatica*	Cape golden mole	-
		Echinops telfairi	Lesser hedgehog tenrec	-
	Tubulidentata	*Orycteropus afer*	Aardvark	**D2B**
	Proboscidea	*Loxodonta africana*	African bush elephant	-
	Sirenia	*Trichechus manatus*	West Indian manatee	**D1**
	Cingulata	*Dasypus novemcinctus*	Nine-banded armadillo	**D2B**
	Scandentia	*Tupaia belangeri*	Northern treeshrew	**D2B**
	Primates	*Otolemur garnettii*	Northern greater galago	-
		Callithrix jacchus	Common marmoset	**D2B, D3**
		Saimiri boliviensis	Black-capped squirrel monkey	-
		Papio anubis	Olive baboon	**D2B, D3**
		Chlorocebus sabaeus	Green monkey	**D2B, D3**
		Macaca fascicularis	Crab-eating macaque	**D2B, D3**
		Macaca mulatta	Rhesus macaque	**D2B, D3**
		Nomascus leucogenys	Northern white-cheeked gibbon	**D2B, D3**
		Pongo abelii	Sumatran orangutan	**D2B, D3**
		Gorilla gorilla	Western gorilla	**D2B, D3**
		Pan paniscus	Bonobo	**D2B, D3**
		Pan troglodytes	Common chimpanzee	**D2B, D3**
		Homo sapiens	Human	**D2B, D3**
	Lagomorpha	*Ochotona princeps*	American pika	-
		Oryctolagus cuniculus	European rabbit	-
	Rodentia	*Mus musculus*	House mouse	-
		Cricetulus griseus	Chinese hamster	-
		Peromyscus maniculatus	North American deermouse	-
		Rattus norvegicus	Brown rat	-
		Chinchilla lanigera	Long-tailed chinchilla	-
		Ictidomys tridecemlineatus	Thirteen-lined ground squirrel	-
		Microtus ochrogaster	Prairie vole	-
		Cavia porcellus	Guinea pig	-
		Jaculus jaculus	Lesser Egyptian jerboa	-
	Eulipotyphla	*Erinaceus europaeus*	European hedgehog	**D2A**
		Condylura cristata	Star-nosed mole	-
		Sorex araneus	Common shrew	**D1**

Class	Order	Species	Common name	RGD position
Mammalia	Cetacea	*Lipotes vexillifer*	Baiji	-
		Balaenoptera acutorostrata	Common minke whale	-
		Physeter catodon	Sperm whale	-
		Tursiops truncatus	Common bottlenose dolphin	-
		Orcinus orca	Killer whale	-
	Artiodactyla	*Sus scrofa*	Wild boar	**D2B**
		Bubalus bubalis	Water buffalo	-
		Bos taurus	Cattle	-
		Vicugna pacos	Alpaca	-
		Camelus ferus	Wild Bactrian camel	-
		Pantholops hodgsonii	Tibetan antelope	-
		Capra hircus	Domestic goat	**D1**
		Ovis aries	Sheep	-
	Chiroptera	*Pteropus alecto*	Black flying fox	**D2A**
		Myotis davidii	David's mouse-eared bat	**D2B**
		Myotis brandtii	Brandt's bat	**D2B**
		Myotis lucifugus	Little brown bat	**D2B**
		Eptesicus fuscus	Big brown bat	**D2B**
	Perissodactyla	*Equus caballus*	Horse	**D2B**
		Ceratotherium simum	White rhinoceros	-
	Carnivora	*Panthera tigris*	Tiger	**D2B**
		Felis catus	Domestic cat	**D2B**
		Canis lupus	Dog	-
		Mustela putorius	European polecat	-
		Odobenus rosmarus	Walrus	-
		Ailuropoda melanoleuca	Giant panda	-
Aves	Galliformes	*Meleagris gallopavo*	Wild turkey	**D3**
		Anas platyrhynchos	Mallard	**D3**
		Gallus Gallus	Red junglefowl	**D3**
	Columbiformes	*Columba livia*	Rock dove	**D3**
	Falconiformes	*Falco cherrug*	Saker falcon	**D3**
		Falco peregrinus	Peregrine falcon	**D3**
	Psittaciformes	*Melopsittacus undulatus*	Budgerigar	**D3**
	Passeriformes	*Geospiza fortis*	Medium ground finch	-
		Ficedula albicollis	Collared flycatcher	-
		Taeniopygia guttata	Zebra finch	-

Figure S1. VE-cadherin structure and evolutionary relationships among organisms. (A) Domain structure of VE-Cadherin indicating the possible positions of RGD motifs. (B) List of VE-Cadherin sequences in mammal and bird species, indicating the number and position of the RGD motifs.

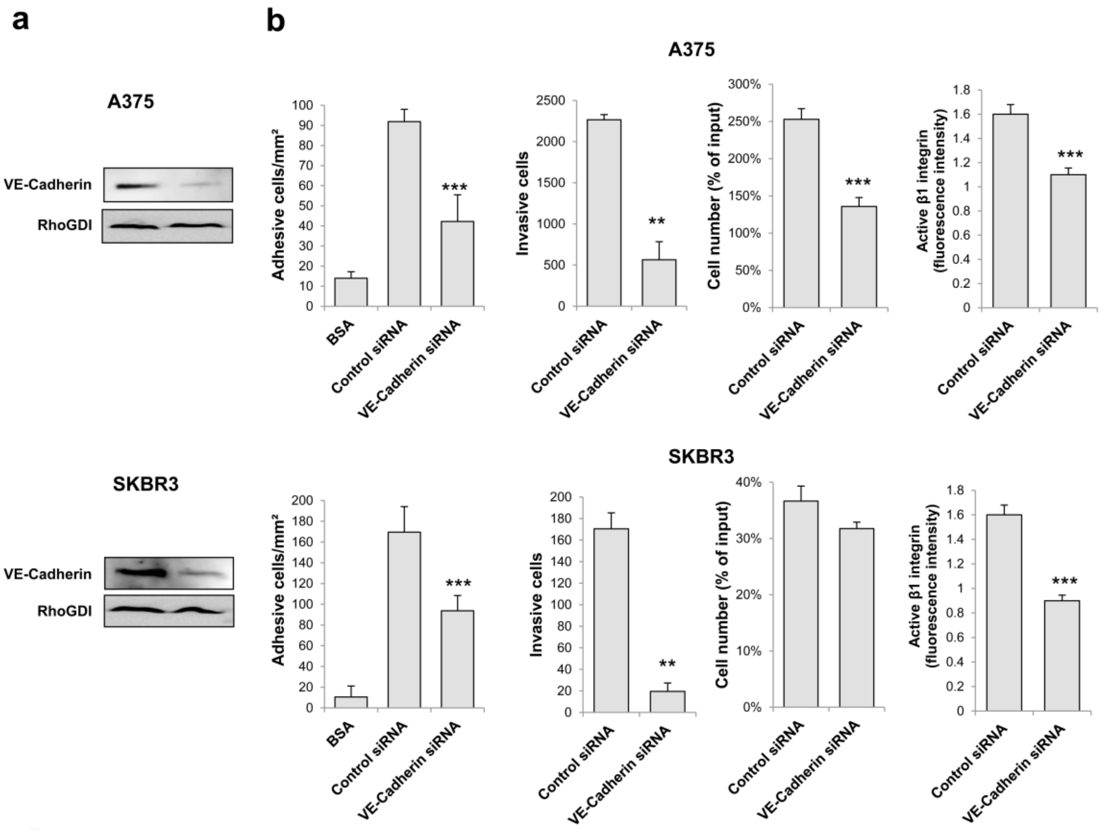

Figure S2. **Silencing of VE-cadherin inhibits cell adhesion, invasion and proliferation in A375 and SKBR3 cell lines**. (a) A375 and SKBR3 cells were transfected with siRNAs against VE-cadherin or with a control siRNA. VE-cadherin expression was assessed by western blot. (b) Transfectants were subjected to adhesion onto Matrigel, invasion through Matrigel and MTT assays to determinate cell proliferation. Cell adhesion, invasion or proliferation was significantly inhibited by VE-cadherin knock-down (** $p < 0.01$, *** $p < 0.001$). (c) Transfectants were analysed by flow cytometry to determine the expression of high-affinity β1 integrin conformation after VE-cadherin silencing.

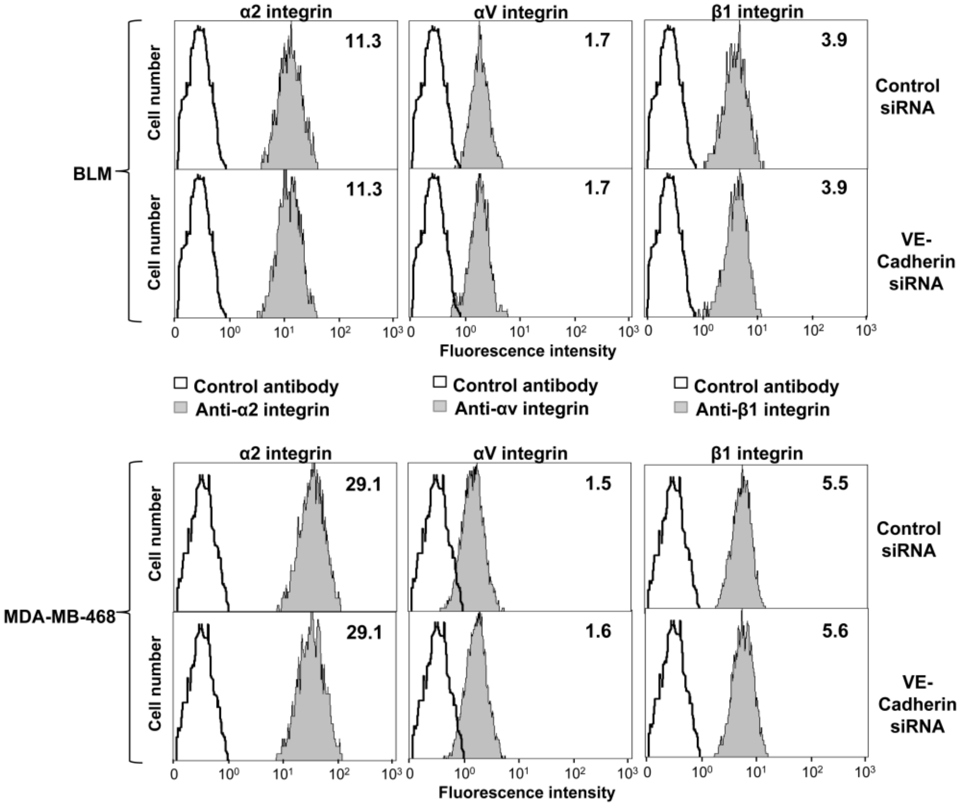

Figure S3. **VE-cadherin silencing does not affect integrin expression on the cell surface**. BLM and MDA-MB-468 cells were transfected with control or VE-Cadherin siRNAs and subjected to flow cytometry assays using the indicated antibodies.

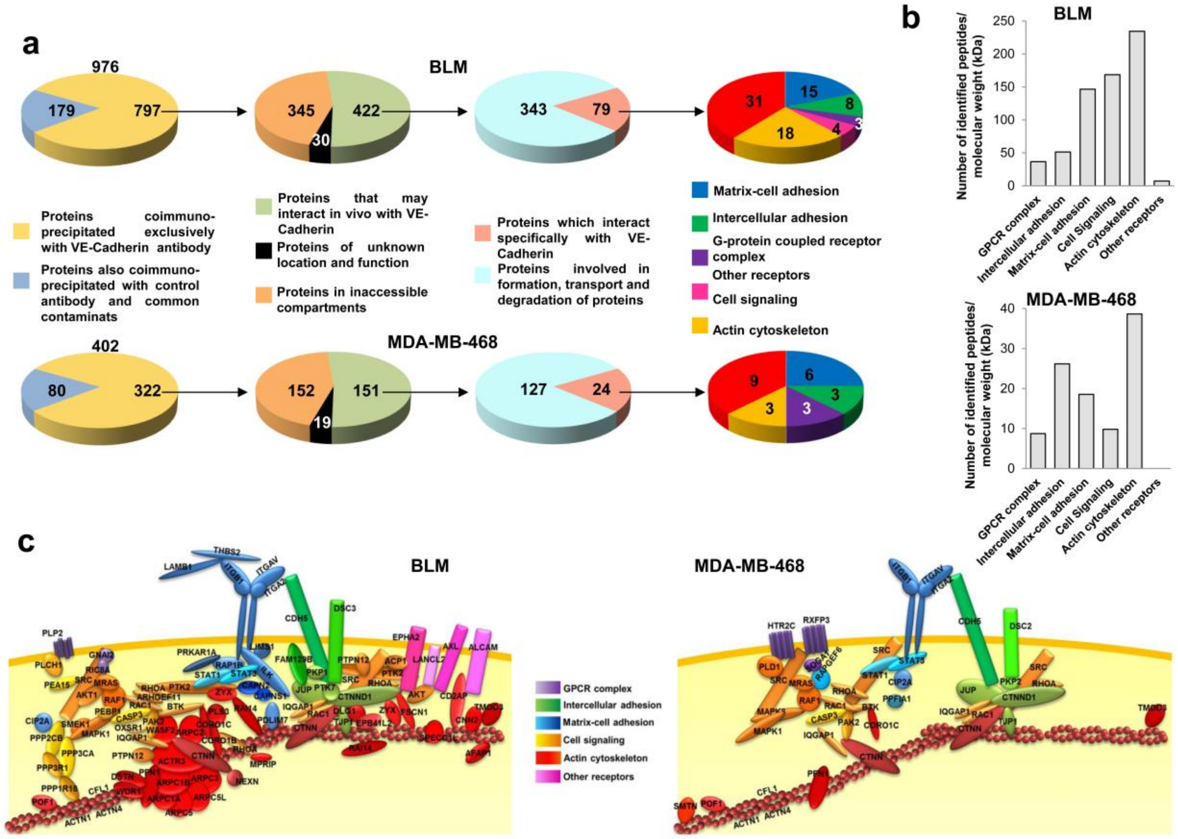

Figure S4. **Gene ontology analysis of proteins co-immunoprecipitated with VE-cadherin**. (A) Classification of VE-cadherin coimmunoprecipitated proteins in BLM and MDA-MB-468 cells after identification by mass spectrometry analysis according to their location and function. Only those proteins that interacted specifically with VE-cadherin were represented in Figure 3a. (B) Gene ontology analysis of the proteins interacting with VE-cadherin. (C) Diagram showing the proteins associated to VE-cadherin as interacting in cis.

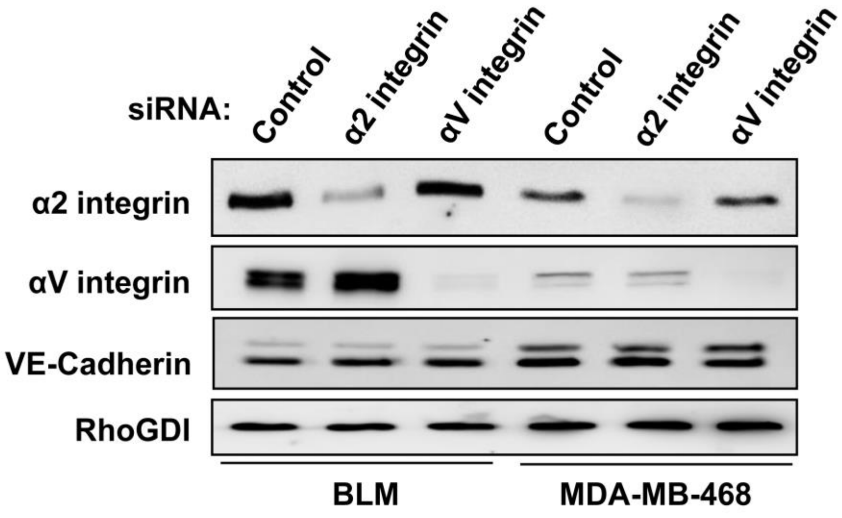

Figure S5. **Silencing of integrin subunits does not affect VE-cadherin expression or the phosphorylation status.** BLM and MDA-MB-468 cells were transfected with the indicated siRNAs, and the cell extracts analyzed by western blot to assess the knock down of the proteins targeted by the siRNAs and its impact on VE-cadherin expression.

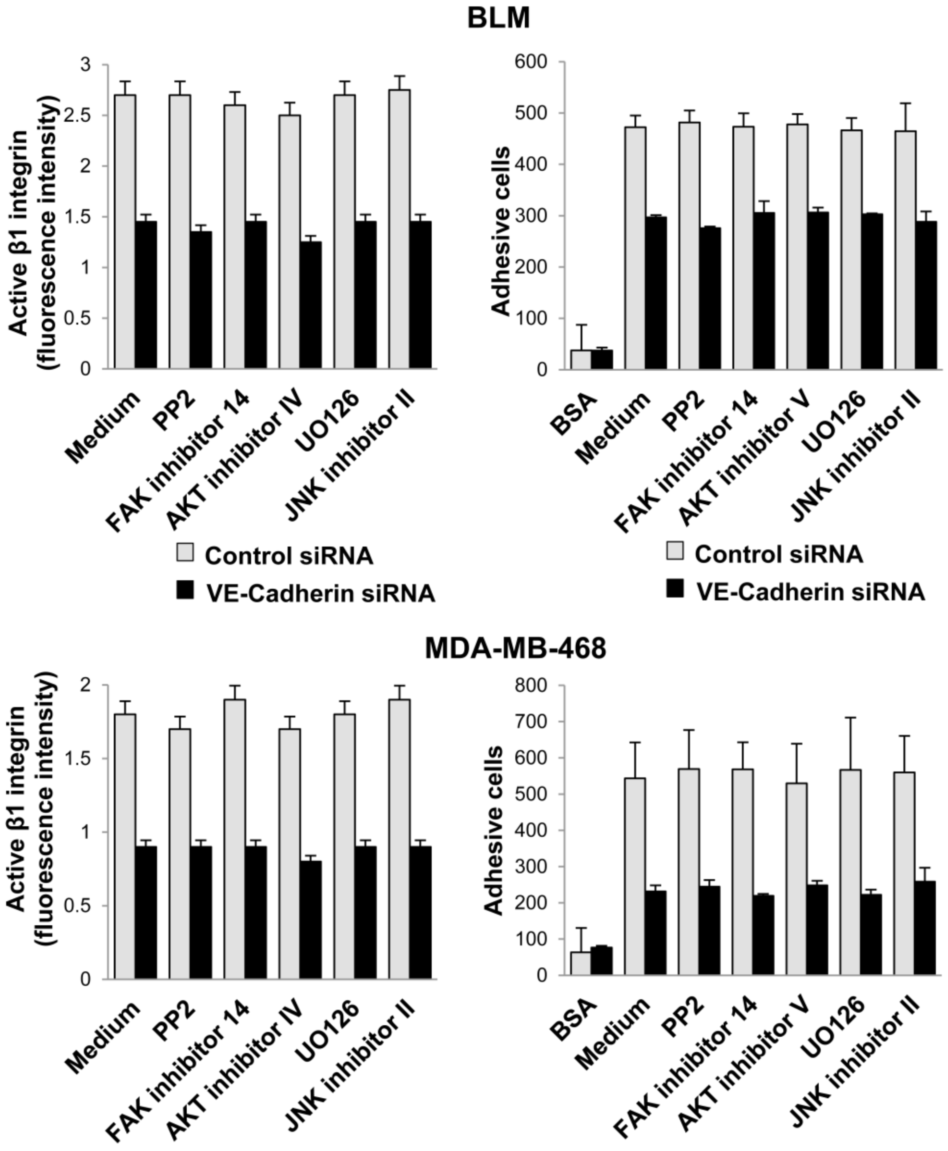

Figure S6. **VE-cadherin induced β1 integrin activation and cell adhesion are independent of intracellular signaling.** BLM and MDA-MB-468 cells were transfected with control or VE-cadherin siRNAs. After 48 h, transfectants were exposed for 2 h to the indicated inhibitors and subjected to flow cytometry assays to assess high affinity conformation of β1 integrin (A) or cell adhesion to Matrigel (B). No effect was observed for any of the inhibitors.
