# Supplementary material for: VE-cadherin RGD motifs promote metastasis and constitute a potential therapeutic target in melanoma and breast cancers
Source: Oncotarget. 2016 Dec 9;8(1):215–27. doi: 10.18632/oncotarget.13832 (PMC5352113; doi:10.18632/oncotarget.13832)
Supplement: Supplementary file 2 [file oncotarget-08-215-s002.docx]

**Table S1. List of antibodies used in the different applications**

| **Antibody** | **Target** | **Application** | **Origin** |
| --- | --- | --- | --- |
| BV9 | VE-Cadherin | flow cytometry, immunoprecipitation, western blotting | Santa Cruz Biotechonogies |
| 441145G | Phospho Y731 VE-Cadherin | western blotting | Invitrogen |
| K-20 | β1-integrin | western blotting | Santa Cruz Biotechonogies |
| HUTS-21 | β1-integrin in high affinity conformation | flow cytometry | BD Transduction Laboratories |
| P1E6 | α2-integrin | flow cytometry, immunoprecipitation, western blotting | Santa Cruz Biotechonogies |
| H-2 | αv-integrin | flow cytometry, immunoprecipitation, western blotting | Santa Cruz Biotechonogies |
| Ab-3 | H-Ras, K-Ras, H-Ras | western blotting | Calbiochem |
| A-20 | RhoGDIα | western blotting | Santa Cruz Biotechonogies |
| B-7 | αTubulin | western blotting | Santa Cruz Biotechonogies |
| E-8 | Caspase-3 | western blotting | Santa Cruz Biotechonogies |
| 540 | Raf-1 | western blotting | Santa Cruz Biotechonogies |
| EP6376 | Cofilin-1 | western blotting | Abcam |
| 26C4 | RhoA | western blotting | Santa Cruz Biotechonogies |
| 102 | Rac1 | western blotting | BD Transduction Laboratories |
| 98/pp120 | p120 Catenin | western blotting | BD Transduction Laboratories |
| ab79218 | PAK | western blotting | Abcam |
| 9272 | AKT | western blotting | Cell signaling Technology |
| D9E | Phospho-Akt (Ser473) | western blotting | Cell signaling Technology |
| AF3389 | Src | western blotting | R&D Systems |
| 2101 | Phospho-Src Family (Tyr416) | western blotting | Cell signaling Technology |
| L34F14 | p44/42 MAP Kinase | western blotting | Cell signaling Technology |
| D13.14.4E | phospho-Thr202-p44/phospho-Tyr 204-p42 MAPK | western blotting | Cell signaling Technology |
| A-17 | FAK | western blotting | Santa Cruz Biotechonogies |
| FAK (pY397) 14 | phospho-Tyr397-FAK | western blotting | BD Transduction Laboratories |
| 56G8 | JNK | western blotting | Cell signaling Technology |
| G9 | phospho-Thr183/Tyr185-JNK | western blotting | Cell signaling Technology |
