# Supplementary material for: VE-cadherin RGD motifs promote metastasis and constitute a potential therapeutic target in melanoma and breast cancers
Source: Oncotarget. 2016 Dec 9;8(1):215–27. doi: 10.18632/oncotarget.13832 (PMC5352113; doi:10.18632/oncotarget.13832)
Supplement: Supplementary file 3 [file oncotarget-08-215-s003.docx]

**Table S2: Proteins coimmunoprecipitated with VE-Cadherin**

| **Accession** | **Name** | **Description** | **BLM** | | | | **MDA-MB-468** | | | |
| --- | --- | --- | --- | --- | --- | --- | --- | --- | --- | --- |
|  |  |  | **Score** | **Coverage** | **# Peptides** | **# PSM** | **Score** | **Coverage** | **# Peptides** | **# PSM** |
| O43707 | ACTN4 | Alpha-actinin-4 | 734,66 | 61,58 | 50 | 188 | 92,38 | 10,65 | 8 | 23 |
| P12814 | ACTN1 | Alpha-actinin-1 | 528,50 | 60,65 | 46 | 133 | 77,31 | 9,42 | 7 | 19 |
| P14923 | JUP | Junction plakoglobin | 113,96 | 22,01 | 15 | 34 | 50,50 | 7,65 | 6 | 17 |
| P05556 | ITGB1 | Integrin beta-1 | 89,03 | 13,41 | 9 | 27 | 29,55 | 3,38 | 3 | 9 |
| P53814 | SMTN | Smoothelin |  |  |  |  | 24,29 | 3,50 | 3 | 8 |
| P07737 | PFN1 | Profilin-1 | 92,38 | 59,29 | 7 | 25 | 15,51 | 18,57 | 2 | 4 |
| P46940 | IQGAP1 | Ras GTPase-activating-like protein IQGAP1 | 242,92 | 24,38 | 33 | 65 | 13,00 | 1,75 | 3 | 4 |
| Q14247 | CTTN | Src substrate cortactin | 33,24 | 11,27 | 5 | 9 | 10,28 | 5,64 | 3 | 3 |
| Q9ULV4 | CORO1C | Coronin-1C | 55,01 | 15,82 | 7 | 17 | 9,44 | 4,64 | 2 | 3 |
| Q99959 | PKP2 | Plakophilin-2 |  |  |  |  | 8,36 | 1,21 | 1 | 3 |
| P40763 | STAT3 | Signal transducer and activator of transcription 3 | 35,16 | 11,46 | 5 | 8 | 6,20 | 1,64 | 1 | 2 |
| Q8TCG1 | CIP2A | Protein CIP2A | 14,75 | 3,22 | 3 | 6 | 5,33 | 2,14 | 2 | 2 |
| Q9NSD7 | RXFP3 | Relaxin-3 receptor 1 |  |  |  |  | 3,59 | 7,56 | 1 | 1 |
| Q13835 | PKP1 | Plakophilin-1 | 8,15 | 3,59 | 2 | 2 | 3,57 | 1,52 | 1 | 1 |
| Q8WVV4 | POF1B | Protein POF1B | 6,20 | 3,57 | 2 | 2 | 3,43 | 1,70 | 1 | 1 |
| Q8TEU7 | RAPGEF6 | Rap guanine nucleotide exchange factor 6 |  |  |  |  | 3,39 | 0,99 | 1 | 1 |
| O94964 | SOGA1 | Protein SOGA1 |  |  |  |  | 3,26 | 2,70 | 1 | 1 |
| P23528 | CFL1 | Cofilin 1 | 48,57 | 42,95 | 6 | 10 | 3,24 | 7,38 | 1 | 1 |
| Q13136 | PPFIA1 | Liprin-alpha-1 |  |  |  |  | 2,99 | 2,82 | 1 | 1 |
| Q06187 | BTK | Truncated Bruton agammaglobulinemia tyrosine kinase | 7,75 | 1,41 | 1 | 3 | 2,70 | 1,41 | 1 | 1 |
| Q02487 | DSC2 | Desmocollin-2 |  |  |  |  | 2,66 | 1,06 | 1 | 1 |
| P06756 | ITGAV | Integrin alpha-V | 6,22 | 1,00 | 1 | 2 | 2,60 | 0,80 | 1 | 1 |
| Q13393 | PLD1 | Phospholipase D1 |  |  |  |  | 2,08 | 5,63 | 1 | 1 |
| P28335 | HTR2C | 5-hydroxytryptamine receptor 2C |  |  |  |  | 2,03 | 1,75 | 1 | 1 |
| Q07157 | TJP1 | Tight junction protein ZO-1 | 62,58 | 8,33 | 12 | 19 |  |  |  |  |
| Q69YQ0 | SPECC1L | Cytospin-A | 35,51 | 7,42 | 6 | 10 |  |  |  |  |
| P30086 | PEBP1 | Phosphatidylethanolamine-binding protein 1 | 34,44 | 30,48 | 4 | 8 |  |  |  |  |
| P13797 | PLS3 | Plastin-3 | 24,00 | 12,48 | 6 | 7 |  |  |  |  |
| P04899 | GNAI2 | Guanine nucleotide-binding protein G(i) subunit alpha-2 | 22,43 | 13,52 | 4 | 7 |  |  |  |  |
| O60716 | CTNND1 | Catenin delta-1 | 21,96 | 9,62 | 6 | 7 |  |  |  |  |
| P04632 | CAPNS1 | Calpain small subunit 1 | 20,92 | 23,93 | 2 | 4 |  |  |  |  |
| P07996 | THBS1 | Thrombospondin-1 | 20,64 | 5,62 | 5 | 6 |  |  |  |  |
| Q9NR12 | PDLIM7 | PDZ and LIM domain protein 7 | 18,04 | 4,60 | 2 | 6 |  |  |  |  |
| Q9BR76 | CORO1B | Coronin-1B | 17,25 | 5,93 | 3 | 6 |  |  |  |  |
| P42224 | STAT1 | Signal transducer and activator of transcription 1-alpha/beta | 16,33 | 4,92 | 3 | 5 |  |  |  |  |
| P62714 | PPP2CB | Serine/threonine-protein phosphatase 2A catalytic subunit beta | 16,01 | 10,68 | 3 | 5 |  |  |  |  |
| Q13740 | ALCAM | CD166 antigen | 15,41 | 4,70 | 2 | 4 |  |  |  |  |
| Q9NYL9 | TMOD3 | Tropomodulin-3 | 14,96 | 11,65 | 3 | 4 |  |  |  |  |

| **Accession** | **Name** | **Description** | **BLM** | | | | **MDA-MB-468** | | | |
| --- | --- | --- | --- | --- | --- | --- | --- | --- | --- | --- |
|  |  |  | **Score** | **Coverage** | **# Peptides** | **# PSM** | **Score** | **Coverage** | **# Peptides** | **# PSM** |
| Q15942 | ZYX | Zyxin | 14,74 | 8,70 | 3 | 5 |  |  |  |  |
| P10644 | PRKAR1A | cAMP-dependent protein kinase type I-alpha regulatory subunit | 14,55 | 4,99 | 2 | 5 |  |  |  |  |
| P17655 | CAPN2 | Calpain-2 catalytic subunit | 13,71 | 4,18 | 3 | 5 |  |  |  |  |
| P60981 | DSTN | Destrin | 13,25 | 21,62 | 3 | 4 |  |  |  |  |
| P48059 | LIMS1 | LIM and senescent cell antigen-like-containing domain protein 1 | 11,99 | 3,69 | 1 | 3 |  |  |  |  |
| Q9BPX5 | ARPC5L | Actin-related protein 2/3 complex subunit 5-like protein | 11,11 | 16,34 | 2 | 3 |  |  |  |  |
| Q8N556 | AFAP1 | Actin filament-associated protein 1 | 11,07 | 2,47 | 2 | 4 |  |  |  |  |
| Q04941 | PLP2 | Proteolipid protein 2 | 10,86 | 8,55 | 1 | 3 |  |  |  |  |
| Q9NPQ8 | RIC8A | Synembryn-A | 10,75 | 6,86 | 4 | 4 |  |  |  |  |
| Q15121 | PEA15 | Astrocytic phosphoprotein PEA-15 | 10,14 | 9,26 | 1 | 3 |  |  |  |  |
| P04049 | RAF1 | RAF proto-oncogene serine/threonine-protein kinase | 9,81 | 4,55 | 2 | 3 |  |  |  |  |
| P24666 | ACP1 | Low molecular weight phosphotyrosine protein phosphatase | 8,85 | 12,86 | 1 | 3 |  |  |  |  |
| O95747 | OXSR1 | Serine/threonine-protein kinase OSR1 | 8,81 | 4,91 | 2 | 3 |  |  |  |  |
| Q6IN85 | SMEK1 | Serine/threonine-protein phosphatase 4 regulatory subunit 3A | 8,60 | 5,37 | 3 | 3 |  |  |  |  |
| P61224 | RAP1B | Ras-related protein Rap-1b | 8,43 | 43,75 | 2 | 3 |  |  |  |  |
| O15143 | ARPC1B | Actin-related protein 2/3 complex subunit 1B | 8,11 | 3,49 | 1 | 2 |  |  |  |  |
| O15144 | ARPC2 | Actin-related protein 2/3 complex subunit 2 | 8,06 | 9,67 | 3 | 3 |  |  |  |  |
| Q13177 | PAK2 | Serine/threonine-protein kinase PAK 2 | 8,03 | 5,73 | 3 | 3 |  |  |  |  |
| P63098 | PPP3R1 | Calcineurin subunit B type 1 | 7,07 | 7,50 | 1 | 2 |  |  |  |  |
| Q16658 | FSCN1 | Fascin | 6,92 | 5,07 | 2 | 2 |  |  |  |  |
| Q9NS86 | LANCL2 | LanC-like protein 2 | 6,91 | 2,44 | 1 | 2 |  |  |  |  |
| Q6WCQ1 | MPRIP | Myosin phosphatase Rho-interacting protein | 6,84 | 1,78 | 1 | 2 |  |  |  |  |
| O15511 | ARPC5 | Actin-related protein 2/3 complex subunit 5 | 6,79 | 7,95 | 1 | 2 |  |  |  |  |
| P61158 | ACTR3 | Actin-related protein 3 | 6,35 | 5,45 | 2 | 2 |  |  |  |  |
| Q92747 | ARPC1A | Actin-related protein 2/3 complex subunit 1A | 6,31 | 2,70 | 1 | 2 |  |  |  |  |
| P28482 | MAPK1 | Mitogen-activated protein kinase 1 | 6,22 | 2,85 | 1 | 2 |  |  |  |  |
| Q9P0K7 | RAI14 | Ankycorbin | 5,82 | 2,10 | 2 | 2 |  |  |  |  |
| Q6FHE4 | CNN2 | Calponin-2 | 5,32 | 7,43 | 1 | 2 |  |  |  |  |
| Q0ZGT2 | NEXN | Nexilin | 5,27 | 2,49 | 1 | 2 |  |  |  |  |
| O15145 | ARPC3 | Actin-related protein 2/3 complex subunit 3 | 4,60 | 15,48 | 1 | 1 |  |  |  |  |
| P29317 | EPHA2 | Ephrin type-A receptor 2 | 4,10 | 1,23 | 1 | 1 |  |  |  |  |
| Q86SQ0 | PHLDB2 | Pleckstrin homology-like domain family B member 2 | 3,92 | 2,07 | 1 | 1 |  |  |  |  |
| P17301 | ITGA2 | Integrin alpha-2 | 3,76 | 1,56 | 1 | 1 |  |  |  |  |

| **Accession** | **Name** | **Description** | **BLM** | | | | **MDA-MB-468** | | | |
| --- | --- | --- | --- | --- | --- | --- | --- | --- | --- | --- |
|  |  |  | **Score** | **Coverage** | **# Peptides** | **# PSM** | **Score** | **Coverage** | **# Peptides** | **# PSM** |
| Q6NYC8 | PPP1R18 | Phostensin | 3,64 | 2,77 | 1 | 1 |  |  |  |  |
| O43491 | EPB41L2 | Band 4.1-like protein 2 | 3,55 | 9,32 | 1 | 1 |  |  |  |  |
| P42574 | CASP3 | Caspase-3 | 3,33 | 9,40 | 1 | 1 |  |  |  |  |
| P07942 | LAMB1 | Laminin subunit beta-1 | 3,32 | 0,62 | 1 | 1 |  |  |  |  |
| O15085 | ARHGEF11 | Rho guanine nucleotide exchange factor 11 | 3,15 | 0,72 | 1 | 1 |  |  |  |  |
| Q12959 | DLG1 | Disks large homolog 1 | 3,14 | 1,52 | 1 | 1 |  |  |  |  |
| P30530 | AXL | Tyrosine-protein kinase receptor UFO | 3,11 | 1,76 | 1 | 1 |  |  |  |  |
| Q14574 | DSC3 | Desmocollin-3 | 3,10 | 1,07 | 1 | 1 |  |  |  |  |
| Q9Y5K6 | CD2AP | CD2-associated protein | 2,98 | 1,56 | 1 | 1 |  |  |  |  |
| Q05209 | PTPN12 | Tyrosine-protein phosphatase non-receptor type 12 | 2,96 | 1,54 | 1 | 1 |  |  |  |  |
| Q4KWH8 | PLCH1 | 1-phosphatidylinositol 4,5-bisphosphate phosphodiesterase eta-1 | 2,70 | 0,80 | 1 | 1 |  |  |  |  |
| Q13418 | ILK | Integrin-linked protein kinase | 2,58 | 2,20 | 1 | 1 |  |  |  |  |
| Q9Y6W5 | WASF2 | Wiskott-Aldrich syndrome protein family member 2 | 2,34 | 1,61 | 1 | 1 |  |  |  |  |
| Q08209 | PPP3CA | Serine/threonine-protein phosphatase | 2,30 | 2,60 | 1 | 1 |  |  |  |  |
| Q13308 | PTK7 | Inactive tyrosine-protein kinase 7 | 2,27 | 3,50 | 1 | 1 |  |  |  |  |
| O75083 | WDR1 | WD repeat-containing protein 1 | 2,07 | 3,27 | 1 | 1 |  |  |  |  |
